# Leader-Based Flocking of Multiple Swarm Robots in Underwater Environments

**DOI:** 10.3390/s23115305

**Published:** 2023-06-02

**Authors:** Jonghoek Kim

**Affiliations:** System Engineering Department, Sejong University, Seoul 05006, Republic of Korea; jonghoek@gmail.com

**Keywords:** networked system, underwater sensor networks, flocking control, communication connectivity, underwater cluttered environment, local interaction, leader-based controls

## Abstract

Considering underwater environments, this paper tackles flocking of multiple swarm robots utilizing one leader. The mission of swarm robots is to reach their goal while not colliding with a priori unknown 3D obstacles. In addition, the communication link among the robots needs to be preserved during the maneuver. Only the leader has sensors for localizing itself while accessing the global goal position. Every robot, except for the leader, can measure the relative position and the ID of its neighboring robots by utilizing proximity sensors such as Ultra-Short BaseLine acoustic positioning (USBL) sensors. Under the proposed flocking controls, multiple robots flock inside a 3D virtual sphere while preserving communication connectivity with the leader. If necessary, all robots rendezvous at the leader to increase connectivity among the robots. The leader herds all robots to reach the goal safely, while the network connectivity is maintained in cluttered underwater environments. To the best of our knowledge, our article is novel in developing underwater flocking controls utilizing one leader, so that a swarm of robots can safely flock to the goal in a priori unknown cluttered environments. MATLAB simulations were utilized to validate the proposed flocking controls in underwater environments with many obstacles.

## 1. Introduction

This paper tackles flocking of multiple swarm robots utilizing one leader. Here, a flocking maneuver indicates that the velocity of each robot matches that of the leader [1]. Research on flocking behaviour stems from the coordination phenomena in nature, from the swarming of bacteria and biochemical cellular networks to the flocking of birds, schooling of fish, and herding of land animals.

In our paper, the mission of the underwater swarm robots is to reach their goal while not colliding with a priori unknown 3D obstacles. In addition, the communication link among the robots needs to be preserved during the maneuvering of the swarm robots.

Unmanned Underwater Vehicles (UUVs) can be divided into two categories based on whether their bodies are streamlined. The UUV’s shape is determined by application requirements. For instance, a streamlined shape reduces water resistance and is desirable if the robot needs to move at high speeds. However, if underwater detection or operation tasks are the robot’s primary roles, then a non-streamlined shape can be desirable.

Due to the high water pressure resistance of spherical objects, spherical UUVs can perform rotational motions with a 0 degree turn radius. Various spherical UUVs have been developed thus far [2,3,4,5,6]. References [2,4,5,6,7] addressed a spherical UUV with hybrid propulsion devices, including vectored water-jet and propeller thrusters. The three Degree-of-Freedom (DoF) motions, including surging, heaving, and yawing, were performed in a swimming pool. References [2,5,7] further demonstrated that by adopting vectored water-jets, a spherical UUV can be made to maneuver freely in any direction. Considering a spherical UUV, ref. [8] utilized fuzzy proportional–integral–derivative (PID) controls to independently control the robot’s maneuver in all directions. Since spherical UUVs are highly maneuverable, our article considers a spherical UUV [2,3,4,5,6] as our robot platform.

If we consider an agent maneuvering in known environments, then it can plan a safe path from the start to the goal utilizing various planning methods, such as those in [9,10,11,12,13]. However, underwater cluttered environments increase the difficulty of controlling multiple robots.

Our paper considers an a priori unknown cluttered environment such that obstacle information is not known in advance. An underwater robot can only detect its nearby obstacle utilizing its local sensors, which include active sonars, with a finite sensing range. In this case, a robot’s collision avoidance control is reactive since the control relies on the robot’s local sensors. Since a robot maneuvers in underwater cluttered environments, obstacles can hinder a robot’s maneuver or block the communication link among robots.

Our paper considers a case where all robots are deployed in unknown cluttered underwater environments where an external localization system, such as Global Positioning Systems (GPS), is not accessible. Many papers on multi-agent controls [14,15,16,17,18] assumed that each robot is localized in the workspace. However, robot localization is not trivial in underwater environments since GPS is not available.

This paper handles a practical scenario where multiple robots are located in cluttered underwater environments where GPS is not available. We address flocking controls to make all robots flock inside a 3D virtual sphere such that the network connectivity is maintained during the maneuver.

Only one robot, called the *leader*, can access the global coordinates of the goal point. Furthermore, only the leader can localize itself utilizing various localization approaches. For instance, the leader can use various sensors, such as FOG-IMU (Fiber Optic Gyro-Inertial Measurement Units) in [19], for improving its localization performance. In addition, the leader can use Visual-Inertial Simultaneous Localization And Mapping (VI-SLAM) in [20,21,22]. Here, VI-SLAM integrates an imaging sensor and an Inertial Measurement Unit (IMU) for the leader’s localization.

On the other hand, a *follower*, a robot that is not a leader, has an IMU and can locate itself for a single sampling interval. This one-step localization is feasible since localization error under an IMU integrates as time goes on. Every follower can measure the relative position and ID of its neighboring robots by utilizing its proximity sensors. It is assumed that the maximum sensing range of a proximity sensor is bounded above. Furthermore, each follower can utilize Ultra-Short BaseLine acoustic positioning (USBL) sensors to measure the relative position and the ID of its neighbors.

Only the leader can localize itself while accessing the global goal position. Since the leader can locate itself while accessing the goal position, the leader herds all followers to a predefined goal while preserving communication connectivity in 3D cluttered environments. Under the proposed flocking controls, multiple robots flock inside a 3D virtual sphere while preserving communication connectivity with the leader. If necessary, all robots rendezvous at the leader to increase the connectivity among robots.

We introduce the proposed 3D flocking controls briefly. Let $L^{R}$ denote the maximum communication-sensing range of the leader. Additionally, let $F^{R}$ denote the maximum communication-sensing range of a follower. Note that $L^{R}\geq F^{R}$ since the leader is superior to other followers. The formation of the swarm robots is maintained such that all followers are within the virtual sphere with radius $F^{R}\times \eta$ centered at the leader. Here, η≤1 is a tuning constant. As we decrease η, we generate smaller swarm formation. In this way, the leader can sense and communicate with every follower within one hop link. The velocity command of the leader is transmitted to all followers so that the velocity of each follower matches that of the leader. In this way, flocking behavior can be performed by all robots within $\frac{F^{R}}{C}$ seconds. Here, *C* represents the signal speed.

Note that the formation of the swarm robots is maintained such that the leader can communicate with every follower within one hop link. This one hop communication is desirable since multi-hop communication can lead to a delay in velocity matching. In our paper, velocity matching is feasible since the leader can communicate with every follower within one hop link.

In practice, there exists some motion perturbation, such as sea current, for each robot. Due to motion perturbation or obstacles, a follower may get out of the virtual sphere with radius $F^{R}\times \eta$ centered at the leader. In addition, sudden obstacles may block the communication link between a follower and the leader. In these cases, we apply 3D distributed gathering controls in Section 4.2 so that every follower will rendezvous with the leader until all followers are within the virtual sphere with radius $F^{R}\times \eta$. Once every follower is sufficiently close to the leader, a flocking maneuver is performed by all the robots.

The leader approaches the goal while avoiding a collision with the obstacles. Whenever the leader detects a nearby obstacle by utilizing its local sensors, it changes its heading for collision avoidance. Reactive collision avoidance is utilized to avoid a collision with an a priori unknown obstacle. The safety margin of the leader is set so that all robots inside the 3D virtual sphere centered at the leader can achieve collision avoidance. In this way, all robots can reach the goal safely while maintaining communication connectivity in cluttered 3D environments.

To the best of our knowledge, our article is novel in developing 3D flocking controls based on a single leader, so that a swarm of robots can safely flock to its goal in a priori unknown cluttered underwater environments. MATLAB simulations were utilized to validate the proposed flocking controls in underwater environments with many obstacles.

Our article is organized as follows. Section 2 reviews the literature related to our paper. Section 3 introduces definitions and assumptions utilized in our article. Section 4 introduces the flocking control proposed in our paper. Section 5 introduces MATLAB simulation results. Conclusion are discussed in Section 6.

## 2. Literature Review

Considering 2D environments, many papers discussed how to make multiple robots rendezvous utilizing distributed controls [23,24,25,26]. Reference [27] addressed event-triggered rendezvous controls to make every robot rendezvous while reducing communication resources. However, refs. [27,28] did not consider the limited communication/sensing range of each robot. We propose 3D flocking controls while considering the limited communication/sensing range of each robot.

References [25,29,30] addressed distributed rendezvous controls, which are based on local interaction within a limited range. If we consider an a priori known goal position as a rendezvous point, then we can utilize rendezvous controls to make all agents rendezvous at the goal. However, in our paper, the goal may not be detected by an agent since the goal can be too far from all robots. In addition, a flocking maneuver (velocity matching) cannot be performed as we apply rendezvous controls only. Note that a velocity matching approach [1] is required to achieve a flocking maneuver from all the robots. Therefore, our paper is distinct from other papers based on distributed rendezvous controls, for example [23,24,25,26,27,28].

Considering 2D environments, many papers have considered the formation control of multiple robots [31,32,33,34,35]. Considering a cluttered 2D environment that is known in advance, ref. [36] developed a discrete-event coordination strategy that plans collision-free transitions between arbitrarily defined formations. The authors of [36] assumed the existence of a centralized supervisory controller that knows the entire environment and can communicate with all agents without any restrictions. However, this assumption is too strong in practice. The authors of [35] developed a heading-independent relative localization and tracking method for indoor leader–follower flight. The authors of [35] considered the 2D space, where each robot maneuvers while maintaining its height. The authors of [35] did not consider the fact that the communication link among robots may be blocked in environments with many obstacles. The authors of [34] introduced a system architecture enabling end-to-end connectivity. The aim of [34] was assuring communication connectivity while the leader performs its mission. The authors of [34] assumed that the obstacle environment is known in advance, which may not be feasible in practice.

Our paper is distinct from the papers in the previous paragraph since we consider an a priori unknown cluttered 3D environment. We further propose 3D flocking controls, which consider the limited communication/sensing range of each robot explicitly.

Considering 2D environments, ref. [31,37,38] introduced splitting and merging strategies for a team of networked robots navigating in formation while avoiding obstacles. However, one can argue that a splitting maneuver is not desirable since it can result in the communication loss of an agent. In other words, a robot may be disconnected from the leader during a splitting maneuver. Our paper is distinct from [31,32,33,34,35] since our multi-agent controls consider flocking maneuvers in 3D cluttered environments, which are not known in advance.

Reference [39] addressed guidance of distributed robots using one leader. Reference [39] used rendezvous controls in [40] to make all robots rendezvous with the leader. Once they rendezvous, the leader guides all robots towards the goal. In [39,40], all robots rendezvoused with the leader by generating the adjacency matrix. Thereafter, a tree, which is rooted with the leader, is built based on the adjacency matrix. The tree is then used to make all robots rendezvous with the leader. In our paper, all robots rendezvous with the leader by generating a tree rooted with the leader. We use the distributed Breadth First Search (BFS) method in [41] for distributed tree generation. The BFS method has the computational complexity of O(1), and we do not require generation of the adjacency matrix, as in [39].

In [39], the leader’s 3D control for reaching the goal had singularity. In our paper, the leader is controlled on a vertical plane, which contains both the leader and the goal. In this way, the 3D path plan is reduced to a simple 2D path plan on the plane. Moreover, ref. [39] did not address the flocking controls of multiple robots. Note that flocking maneuver indicates that the velocity of each robot matches that of the leader [1]. We address the flocking maneuvers of all robots in a 3D cluttered environments.

Flocking maneuver implies that the velocity of each robot matches that of the leader [1]. Any kind of velocity-matching rule term in control algorithms should satisfy the following assumption: it should be local while relaxing the velocity difference of robots close to each other [18]. For instance, refs. [18,38] addressed distributed velocity-matching algorithms that contain a viscous friction-like term, aligning the velocities of neighboring agents parallel to each other. References [18,38] did not consider the fact that the sensing communication range of a robot can be bounded, as robots move in unknown cluttered environments. In addition, obstacles in cluttered environments can easily block the link among robots.

In practice, multi-hop communication among robots can lead to a severe delay in velocity matching. In our paper, velocity matching within one hop link becomes feasible since we control the swarm formation so that the leader can communicate with every follower within one hop link. If necessary, all robots rendezvous with the leader to increase the connectivity among robots, based on the gathering algorithms in Section 4.2.

As far as we know, our article is novel in developing 3D flocking controls based on a single leader so that swarm robots can safely flock to the goal in a priori unknown cluttered environments. MATLAB simulations validate the proposed flocking controls in underwater environments with many obstacles.

## 3. Assumptions and Definitions

In our paper, the mission of underwater swarm robots is as follows: *Spherical underwater robots reach their goal while not colliding with a priori unknown 3D obstacles. In addition, the communication link among the robots must be preserved during the maneuver.*

We address several assumptions and definitions in our article. In total, *N* robots are deployed. Let $u_{j}$ (j∈{1,2,⋯,N}) denote the *j*-th robot. Let $u_{N}$ denote the *leader*, whose role is to herd every robot towards the goal while not colliding in a 3D cluttered workspace. Only the leader can access the goal while locating itself during the maneuver. All other robots, except for $u_{N}$, are called *followers*.

This paper considers discrete-time systems. Let dt denote the sampling duration in discrete-time systems. For any variable a, a(k) denotes a at sampling-instant *k*.

In the inertial frame, let $u_{i}\in R^{3}$ denote the 3D coordinates of a robot $u_{i}$ (i∈{1,2,⋯,N}). Let $u_{i}\left(k\right)$ denote $u_{i}$ at sampling-instant *k*. In the inertial frame, the process model of $u_{i}$ is

(1) $$\begin{array}{cc} u_{i}\left(k+1\right) & =u_{i}\left(k\right)+dt\times s_{i}\left(k\right)\times c_{i}\left(k\right). \end{array}$$

The speed of $u_{i}$ at sampling-instant *k* is $s_{i}\left(k\right)$. In practice, a robot moves with bounded speed. Therefore, let $S_{i}$ indicate the maximum speed of $u_{i}$, i.e., $s_{i}\left(k\right)\leq S_{i}$ for all *k*. In (1), $c_{i}\left(k\right)$ is the unit vector indicating the heading direction of $u_{i}$ at sampling-instant *k*. In (1), $s_{i}\left(k\right)\times c_{i}\left(k\right)$ represents the velocity vector of $u_{i}$ at sampling-instant *k*. The process model in (1) has been widely utilized in multi-robot systems [18,29,38,42,43,44,45,46,47].

This paper considers a spherical UUV. References [2,5,7] showed that by adopting vectored water-jets, a spherical UUV can maneuver freely in any direction. The control of a spherical UUV was addressed in [8]. The authors of [8] proposed a decoupling motion control based on the attitude calculation for an underwater spherical robot. The authors of [8] utilized fuzzy PID controllers to independently control the robot’s movement in all directions. Since a spherical UUV is highly maneuverable, the process model in (1) is feasible.

Considering a follower, $F^{R}$ indicates the maximum sensing range of a proximity sensor. It is assumed that $u_{i}$ can communicate with $u_{j}$ if they can measure the relative position of each other utilizing proximity sensors. In other words, the maximum communication range is not shorter than the maximum sensing range.

We say that two agents are *neighbors* in a case where the following conditions are met:

1. Two agents can measure the relative position of each other utilizing proximity sensors, such as USBL.
2. There is no obstacle blocking the line-of-sight between the two agents.

This neighbor definition is commonly utilized in the literature on distributed rendezvous controls [25,29,30]. Since $F^{R}$ is the maximum sensing communication range of a proximity sensor, the relative distance between two neighbors is less than $F^{R}$.

We consider an a priori unknown cluttered environment. The leader can only detect its nearby obstacles by utilizing local sensors, such as active sonars, with a finite sensing range.

Let $D\in R^{3}$ denote the 3D goal position, which is known to the leader only. While the leader moves to reach D, it can sense nearby obstacles by utilizing local sensors.

Let G=(V(G),E(G)) indicate a graph with vertex set V(G) and edge set E(G). In *G*, V(G) represents each agent, and $\left(u_{i},u_{j}\right)\in E\left(G\right)$ indicates that two agents $u_{i}$ and $u_{j}$ can measure the relative position and the ID of each other utilizing proximity sensors such as USBL sensors. Let $\left(u_{i},u_{j}\right)\in E\left(G\right)$ indicate that agent $u_{j}$ is a *neighbor* of $u_{i}$. $\left(u_{i},u_{j}\right)\in E\left(G\right)$ implies that no obstacle blocks the Line-Of-Sight between $u_{i}$ and $u_{j}$.

A *cycle* is a graph forming a closed chain. A *tree* is a graph that has no cycles in it. In a tree, the leader is chosen as the *root*.

In a tree, there exists only one path from any agent *v* to the leader. Every agent that is not *v* along this path is an *ancestor* of *v*. A *descendant* of *v* is an agent of which *v* is an ancestor. A *spanning treeT* is a tree containing all agents in *G*. In a tree, the *parent* of *v*, say p(v), is the neighbor of *v* such that p(v) exists on the path from *v* to the root. A *child* of *v*, say c(v), is an agent of which *v* is the parent.

Initially, *G* contains a connected path between any agent $u_{i}$ (i∈{1,2,⋯,N−1}) and the leader $u_{N}$. This assumption is called the *initialNetworkAssumption*. This assumption is required since every agent moves based on proximity sensors with limited range $F^{R}$. As far as we know, this initialNetworkAssumption is required for every distributed rendezvous control in the literature [25,29,30].

We say that *gathering* is conducted when the following *gathering condition* is satisfied for all i∈{1,2,...,N−1}.

(2) $$\begin{array}{c} ∥u_{i}-u_{N}∥<F^{R}\times \eta \end{array}$$

where η≤1 is a positive constant. In (2), it is implied that all followers are within the virtual sphere, with radius $F^{R}\times \eta$ centered at the leader. Recall that $F^{R}$ is the sensing/communication range of a follower.

## 4. Flocking Control

We control the swarm robots so that all followers are within the virtual sphere, with radius $F^{R}\times \eta$ centered at the leader. In this way, the leader can sense and communicate with every follower within one hop link. This one hop communication is desirable since multi-hop communication can result in a significant delay in velocity matching. In our paper, velocity matching becomes feasible since the leader can communicate with every follower within one hop link.

In (1), $s_{N}\left(k\right)$ and $c_{N}\left(k\right)$ represent the velocity of the leader. To achieve velocity matching, the leader transmits $s_{N}\left(k\right)$ and $c_{N}\left(k\right)$ to all followers at each sampling-instant *k*. Then, the leader’s velocity is transmitted to every follower within $\frac{F^{R}}{C}$ seconds. We assume that the signal speed *C* is sufficiently fast such that $\frac{F^{R}}{C}<dt$ is satisfied.

Note that a follower can locate itself within one sampling interval, dt seconds. Once a follower receives the leader’s velocity information, the follower sets its speed command to $s_{N}\left(k\right)$ and sets its heading command to $c_{N}\left(k\right)$. Then, the follower moves with velocity $s_{N}\left(k\right)\times c_{N}\left(k\right)$ for dt seconds. In this way, velocity matching is achieved within one sampling interval.

Due to motion perturbation or sudden obstacles, a follower may get out of the virtual sphere centered at the leader. In other words, we may have a case where

(3) $$\begin{array}{c} ∥u_{i}-u_{N}∥>\eta \times F^{R}+\epsilon \end{array}$$

where ϵ≈0 is a positive constant. In this case, distributed gathering controls (Algorithm 2) run to make all followers get closer to the leader, until all followers are sufficiently close to the leader.

While the gathering controls (Algorithms 1 and 2) run, the leader stops moving in order to improve the network connectivity. In other words, the leader stops moving and waits for the gathering of all followers. This stop process is utilized to preserve network connectivity during the maneuver of every agent.
**Algorithm 1** Distributed generation of *T*1:Every agent *u* stores hops(u) (hop distance to the root);2:The leader $u_{N}$ initializes $hops(u_{N})=0$;3:One initially sets hops(u)=∞ where $u\neq u_{N}$;4:Initially, $u_{N}$ sends $hops(u_{N})$ to its neighbors;5:**repeat**6:   *u*← every follower;7:   **if** the follower *u* receives hops(n) from its neighbor, say *n* **then**8:     The follower *u* updates hops(u) under (4);9:     If hops(u) is reset to a new one, then *u* sends the new hops(u) to its neighbors;10:   **end if**11:**until **hops(u)≠∞ for all *u*;

**Algorithm 2** Distributed gathering method
1:While all agents stop, one builds the tree *T* rooted at $u_{N}$ under Algorithm 1;2:
**repeat**
3:   *u*← every follower;4:   **if** *u* is a leaf in *T* **then**5:     *u* travels along a path to $u_{N}$; *u* is not a leaf in *T*6:     **if** *u* meets all its descendants **then**7:        *u* travels along a path to $u_{N}$;8:     **end if**9:   **end if**10:   **if** an agent is disabled or interaction link between two neighbors is disconnected    **then**11:      Re-generate *T* by running Algorithm 1;12:   **end if**13:**until** (2) is satisfied;


### 4.1. Distributed Construction of a Tree *T*

One uses Algorithm 1 for the distributed construction of a tree *T* rooted at the leader. Based on the tree *T*, Algorithm 2 runs to make all agents satisfy (2).

We explain Algorithm 1, which builds *T* rooted at $u_{N}$. Algorithm 1 is based on the distributed BFS method in [41]. See Algorithm 2 in [41].

Algorithm 1 shows a distributed method for generating a tree *T* containing all agents. Every agent *u* stores and updates hops(u), which indicates the hop distance to $u_{N}$. The leader $u_{N}$ sets $hops(u_{N})=0$ initially. In addition, one initially sets hops(u)=∞, where $u\neq u_{N}$.

At the beginning of Algorithm 1, $u_{N}$ sends $hops(u_{N})$ to its neighbors. Suppose an agent *u* receives hops(n) from its neighbor, say *n*. Thereafter, *u* updates hops(n) utilizing

(4) $$\begin{array}{c} hops(u)=min(hops(u),hops(n)+1). \end{array}$$

Based on (4), hops(u) can be reset to hops(n)+1. Whenever hops(u) is reset to hops(n)+1, p(u) is updated to *n*. Whenever hops(u) is reset to hops(n)+1, *u* sends the updated hops(u) to its neighbors. In (4), min(a,b) returns a smaller value between *a* and *b*.

References [41,48] proved that the number of message sent by every agent is 1 in distributed BFS methods. This indicates that Algorithm 1 has the computational complexity O(1).

### 4.2. Distributed Gathering Algorithms

We explain Algorithm 2, which makes all followers satisfy (2). Initially (t=0), all leaf agents begin visiting every follower along the path to the leader $u_{N}$. Here, a follower finds a path to $u_{N}$ utilizing *T*. How to make a follower visit the followers along the path to $u_{N}$ is addressed in Section 4.2.1.

In order to maintain network connectivity, a follower’s maneuver must not disconnect the network. Therefore, not every follower initiates maneuvering until they meet all their descendants in *T*. Thus, every follower stores the agent indexes associated with all its descendants.

Consider a follower, say $u^{\prime }$, having one or more children. Only a single path exists from $u^{\prime }$ to $u_{N}$ in *T*. Once $u^{\prime }$ meets all its descendants, $u^{\prime }$ starts traveling along the path to $u_{N}$ under Alg. 2. As time elapses, $p(u^{\prime })$ meets all its descendants, and $p(u^{\prime })$ starts traveling along the path to $u_{N}$. This process iterates until all followers satisfy (2).

Suppose that an agent is disabled or the interaction link between two neighbors is disconnected by moving obstacles. In this situation, one re-builds *T* so that network connectivity among agents can be re-established. Thereafter, Algorithm 2 re-runs to achieve (2).

#### 4.2.1. Visiting Followers along the Path to the Root, Based on Local Sensors

In Algorithm 2, the only control applied to a follower is traveling along the path, say PATH, to the leader in *T*. This control is a high-level control of every follower.

One next addresses local controls to make one agent travel along PATH utilizing local sensors. Consider the case where PATH consists of an agent set $p_{1}\to p_{2}\to p_{3}\cdots \to p_{end}$ in this order. Here, $p_{end}$ is the root. Let $p_{j}\in R^{3}$ denote the 3D position of $p_{j}$ for convenience.

Suppose that $u_{i}$ reached $p_{j}\in R^{3}$ and that the next agent to visit is $p_{j+1}\in R^{3}$. Since $u_{i}$ reached $p_{j}$, $u_{i}$ detects the relative position of $p_{j+1}$ by utilizing its local sensors.

Then, $u_{i}$ moves towards $p_{j+1}$ by setting

(5) $$\begin{array}{c} c_{i}\left(k\right)=\frac{p_{j+1}-u_{i}\left(k\right)}{∥p_{j+1}-u_{i}\left(k\right)∥} \end{array}$$

as the direction control in (1). Furthermore, the speed $s_{i}\left(k\right)$ in (1) is set as $min(S_{i},\frac{∥p_{j+1}-u_{i}\left(k\right)∥}{dt})$. This implies that in the case where $∥p_{j+1}-u_{i}\left(k\right)∥<S_{i}\times dt$, $u_{i}$ reaches $p_{j+1}$ at sampling-instant k+1.

#### 4.2.2. Convergence Analysis

**Lemma** **1.** *Algorithm 2 is distributed. While a follower $u_{i}$ travels along a path in T, the multi-hop communication link of $u_{i}$ is connected to the leader. In addition, $u_{i}$ avoids collision with static obstacles.*

**Proof.**  A follower $u_{i}$ travels along a path in *T* under Algorithm 2. Let $PATH=p_{1}\to p_{2}\to p_{3}...\to p_{end}$ denote the agents along the path. Here, $p_{end}$ is the leader. After $u_{i}$ meets $p_{j}$ (j≤end−1), $u_{i}$ heads towards $p_{j+1}$.

According to the definition of neighbors, no obstacle blocks the line-of-sight between $p_{j}$ and $p_{j+1}$. Therefore, $u_{i}$ does not collide with static obstacles as it moves from $p_{j}$ to $p_{j+1}$.

Variable $u_{i}$ moves utilizing local sensors since PATH∈G and $\left(u_{i},u_{j}\right)\in E\left(G\right)$ indicates that two agents $u_{i}$ and $u_{j}$ can measure the relative position and the ID of each other utilizing proximity sensors. Since $u_{i}$ moves by utilizing local sensors, Algorithm 2 is distributed.

One proves that $p_{j}$, where j∈{1,2,…,end−1}, initiates maneuvering, only after $u_{i}$ meets $p_{j}$. A follower, other than a leaf, initiates maneuvering only after all its descendants in *T* meet it. Any agent in PATH is an ancestor of $u_{i}$. Therefore, $p_{j}$ initiates maneuvering, only after $u_{i}$ meets $p_{j}$.

One proves that while $u_{i}$ travels along an edge from $p_{j}$ to $p_{j+1}$ (j≤end−1), the communication link of $u_{i}$ is connected to the root. The communication link of $u_{i}$ is connected to $p_{j+1}$ utilizing the definition of an edge E(G). Accordingly, $u_{i}$ is connected to the root during its maneuver. This lemma is proved. □

### 4.3. Leader Control

Once the gathering is finished (see (2)) utilizing distributed gathering algorithms in Section 4.2, all followers are within the virtual sphere with radius $F^{R}\times \eta$ centered at the leader. Once the gathering is finished, the leader approaches the goal while avoiding collision with obstacles.

Once the gathering is finished, (Equation (2) is satisfied for all i∈{1,2,…,N−1}), all followers achieve velocity matching with the leader, which leads to the flocking behavior of all agents. To achieve velocity matching, the leader transmits its velocity information ($s_{N}\left(k\right)$ and $c_{N}\left(k\right)$ in (1)) to all followers at every sampling instant *k*. The leader’s velocity is transmitted to every follower within $\frac{F^{R}}{C}$ seconds. We assume that the signal speed *C* is sufficiently fast such that $\frac{F^{R}}{C}<dt$ is satisfied. Once a follower receives the leader’s velocity information, the follower sets its speed command to $s_{N}\left(k\right)$ and sets its heading command to $c_{N}\left(k\right)$. Then, the follower moves with velocity $s_{N}\left(k\right)\times c_{N}\left(k\right)$ for dt seconds. In this way, velocity matching is achieved within one sampling interval.

However, in practice, there exists some motion perturbation, such as sea current, for each agent. Due to motion perturbation, a follower may get out of the virtual sphere with radius $F^{R}\times \eta$ centered at the leader. In addition, sudden obstacles may block the communication link between the leader and a follower.

Due to motion perturbation or obstacles, a follower may not be able to communicate with the leader within one hop link. In this case, the distributed gathering algorithms from Section 4.2 are re-initiated. While the gathering algorithms run, the leader stops moving and waits for all followers.

#### Leader Maneuvers to Reach the Goal Safely

Whenever the leader detects a nearby obstacle utilizing its local sensors, it changes its heading for collision avoidance. The safety margin of the leader is chosen as $S^{M}=L^{R}+\epsilon$. Here, ϵ>0 is a small constant, which is utilized to set the safety margin $S^{M}$ to be larger than $L^{R}$.

In this way, all followers inside the virtual sphere with radius $F^{R}\times \eta$ can achieve collision avoidance. Therefore, all agents can reach the goal safely while maintaining communication connectivity in cluttered 3D environments.

We next introduce how to control the leader once the gathering is finished, i.e., (2) is satisfied. We assume that the leader can localize itself. The leader is controlled to reach its goal while not colliding with a priori unknown obstacles.

In our paper, the leader is controlled on a vertical plane, which contains both the leader and the goal. In this way, the 3D path plan is reduced to a simple 2D path plan on the plane.

Let *obstacle point* denote a point on the obstacle boundary, which is detected by utilizing the leader’s local sensors. At sampling instant *k*, the leader $u_{N}$ detects an obstacle point, say $C_{N}^{o}\left(k\right)$, that is closest to the leader. The local sensors of the leader can provide the information on $C_{N}^{o}\left(k\right)$ at sampling instant *k*.

Let $r_{N}\left(k\right)$ (i∈{1,2,…,N−1}) be denoted as

(6) $$\begin{array}{c} r_{N}\left(k\right)=u_{N}\left(k\right)-C_{N}^{o}\left(k\right). \end{array}$$

In addition, one uses $r_{N}\left(k\right)=∥r_{N}\left(k\right)∥$.

Recall that $S_{N}$ represents the maximum speed of the leader. Furthermore, recall that (6) addressed $r_{N}\left(k\right)$ and that $r_{N}\left(k\right)=∥r_{N}\left(k\right)∥$. If $r_{N}\left(k\right)\geq S^{M}+S_{N}\times dt$, then the leader heads towards D by setting

(7) $$\begin{array}{c} c_{N}\left(k\right)=\frac{D-u_{N}\left(k\right)}{∥D-u_{N}\left(k\right)∥} \end{array}$$

as the direction control in (1). Furthermore, the speed $s_{N}\left(k\right)$ in (1) is set as the maximum speed $S_{N}$.

Let $k_{o}$ denote the sampling instant such that

(8) $$\begin{array}{c} r_{N}\left(k_{o}-1\right)\geq S^{M}+S_{N}\times dt \end{array}$$

and that

(9) $$\begin{array}{c} r_{N}\left(k_{o}\right)<S^{M}+S_{N}\times dt. \end{array}$$

At this sampling instant $k_{o}$, the leader initiates circling around the obstacle.

The leader circles around the obstacle such that it stays inside a plane, say *vertical plane*, which contains both D and $u_{N}\left(k\right)$. The vertical plane is also perpendicular to the xy plane.

Suppose the vertical plane is perpendicular to a vector, say N. Since the vertical plane is perpendicular to the xy plane, N[3]=0. Here, v[j] denotes the *j*-th element in a vector v.

Let $d=D-u_{N}\left(k\right)$ for convenience. Note that the vertical plane contains d and that N[3]=0. Thus, we have

(10) $$\begin{array}{c} d[1]\times N[1]+d[2]\times N[2]=0. \end{array}$$

This implies that the vertical plane is perpendicular to

(11) $$\begin{array}{c} N=(-d[2],d[1],0). \end{array}$$

Let $r_{C}\left(k\right)$ denote a unit vector that is inside the vertical plane such that $r_{C}\left(k\right)$ is perpendicular to $r_{N}\left(k\right)$. Since $r_{C}\left(k\right)$ is inside the vertical plane, $r_{C}\left(k\right)$ is perpendicular to N. We get two vectors for $r_{C}\left(k\right)$, say $r_{C,1}\left(k\right)$ and $r_{C,2}\left(k\right)$.

**Figure 1:**
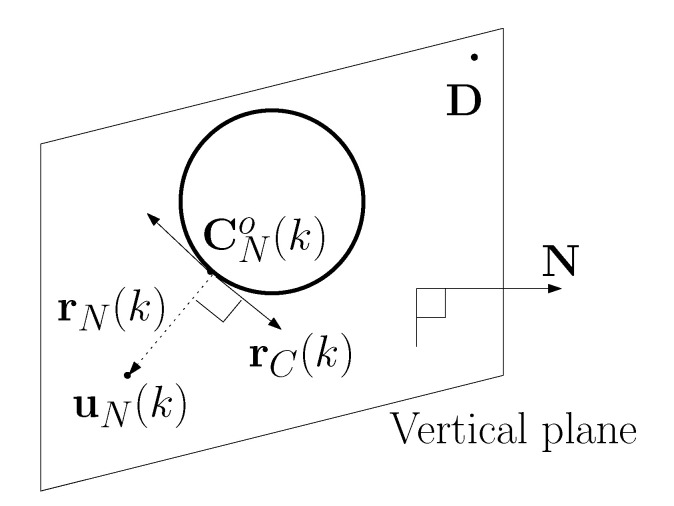
There is a circular obstacle in the vertical plane. The vertical plane is perpendicular to N, and the plane contains both D and $u_{N}\left(k\right)$. In this figure, $r_{N}\left(k\right)$ is plotted with a dotted edge.

See Figure 1 for an illustration. In this figure, there is a circular obstacle in the vertical plane. The vertical plane is perpendicular to N, and the plane contains both D and $u_{N}\left(k\right)$. In this figure, $r_{N}\left(k\right)$ is plotted with a dotted edge.

Since $r_{C}\left(k\right)$ is perpendicular to N in (11), we require that

(12) $$\begin{array}{c} -r_{C,1}\left(k\right)\left[1\right]\times d\left[2\right]+r_{C,1}\left(k\right)\left[2\right]\times d\left[1\right]=0. \end{array}$$

In addition, since $r_{C}\left(k\right)$ is perpendicular to $r_{N}\left(k\right)$, we require that

(13) $$\begin{array}{c} r_{C,1}\left(k\right)\left[1\right]\times r_{N}\left(k\right)\left[1\right]+r_{C,1}\left(k\right)\left[2\right]\times r_{N}\left(k\right)\left[2\right]+r_{C,1}\left(k\right)\left[3\right]\times r_{N}\left(k\right)\left[3\right]=0. \end{array}$$

Based on (12) and (13), we can derive $r_{C}\left(k\right)$ as follows. Recall that we get two vectors for $r_{C}\left(k\right)$, say $r_{C,1}\left(k\right)$ and $r_{C,2}\left(k\right)$. Vector $r_{C,1}\left(k\right)$ is derived as

(14) $$\begin{array}{c} r_{C,1}\left(k\right)=\frac{\left(d[1],d[2],z\right)}{∥(d[1],d[2],z)∥} \end{array}$$

where

(15) $$\begin{array}{c} z=\frac{-d\left[2\right]\times r_{N}\left(k\right)\left[2\right]-d\left[1\right]\times r_{N}\left(k\right)\left[1\right]}{r_{N}\left(k\right)\left[3\right]}. \end{array}$$

Next, $r_{C,2}\left(k\right)$ is derived as

(16) $$\begin{array}{c} r_{C,2}\left(k\right)=-r_{C,1}\left(k\right). \end{array}$$

In order to make the leader circle around an obstacle in the counter-clockwise direction, we find $r_{C}\left(k\right)$, which satisfies

(17) $$\begin{array}{c} N\cdot cross(r_{C}\left(k\right),r_{N}\left(k\right))<0. \end{array}$$

Here, $cross(v_{1},v_{2})$ denotes the cross product of two vectors, say $v_{1}$ and $v_{2}$. In the case where

(18) $$\begin{array}{c} N\cdot cross(r_{C,1}\left(k\right),r_{N}\left(k\right))<0 \end{array}$$

is met, the leader uses

(19) $$\begin{array}{c} c_{N}\left(k\right)=r_{C,1}\left(k\right) \end{array}$$

as the direction control in (1). This indicates that the leader circles around the obstacle in the counter-clockwise direction.

In the case where

(20) $$\begin{array}{c} N\cdot cross(r_{C,2}\left(k\right),r_{N}\left(k\right))<0 \end{array}$$

is met, the leader uses

(21) $$\begin{array}{c} c_{N}\left(k\right)=r_{C,2}\left(k\right) \end{array}$$

as the direction control in (1), since this makes the leader circle around the obstacle in the counter-clockwise direction.

As the leader circles around an obstacle, its speed $s_{N}\left(k\right)$ in (1) is set as the maximum speed $S_{N}$. In practice, we can set $s_{N}\left(k\right)$ as a constant less than $S_{N}$, since circling around an obstacle can be a dangerous maneuver for an agent. For instance, as a human driver drives along a curved lane, he or she slows down to follow the lane carefully.

One may have a case where *z* in (15) is ill-defined, since $r_{N}\left(k\right)\left[3\right]=0$. In this case, we set z=1 in (15), since this *z* satisfies both (12) and (13).

It is desirable that the leader travels along the shortest path to the goal if possible. Recall that $k_{o}$ is the sampling instant when the leader initiates circling around the obstacle. While the leader follows the obstacle boundary utilizing (19) or (21), it detects the moment when both

(22) $$\begin{array}{c} r_{N}\left(k\right)\cdot \left(u_{N}\left(k\right)-D\right)<0 \end{array}$$

and

(23) $$\begin{array}{c} ∥u_{N}\left(k\right)-D∥<∥u_{N}\left(k_{o}\right)-D∥ \end{array}$$

are satisfied. This implies that the obstacle boundary, which the leader has been following, is opposite to the goal direction. At this moment, the leader initiates moving toward the goal utilizing (7), since the leader does not have to keep following the obstacle boundary.

It is desirable that the leader moves towards D if it does not have to circle around the obstacle anymore. Let $Line(u_{N}\left(k\right),D)$ denote the straight line segment connecting the leader $u_{N}\left(k\right)$ and D. Suppose that the relative distance between $Line(u_{N}\left(k\right),D)$ and an obstacle is larger than $S^{M}+S_{N}\times dt$. Then, the leader heads towards D directly under (7).

If $r_{N}\left(k\right)<F^{R}\times \eta$, then the leader is too close to the obstacle, considering the formation radius $F^{R}\times \eta$. In this case, the leader avoids the obstacle abruptly utilizing

(24) $$\begin{array}{c} c_{N}\left(k\right)=\frac{r_{N}\left(k\right)}{∥r_{N}\left(k\right)∥} \end{array}$$

as the direction control in (1). Furthermore, the speed $s_{N}\left(k\right)$ in (1) is set as the maximum speed $S_{N}$. This maximum speed is desirable since collision avoidance is an imminent task.

## 5. Simulation Results

MATLAB simulations are utilized to verify the proposed flocking controls. The sample interval is dt=1 s. The 3D goal, which is only known to the leader, is located at [200,200,200].

Considering each follower, one sets the maximum sensing communication range as $F^{R}=10$ m. Additionally, we use η=1 in (2). Considering the leader’s sensing-communication range, one uses $L^{R}=20$ m. See that $L^{R}>F^{R}$. Furthermore, we use ϵ=1 m in (3).

The maximum speed of an agent $u_{i}$ is $S_{i}=3$ (∀i∈{1,…N}) m/s. The simulation ends when the leader is within $S_{N}\times dt$ meters from the goal.

Instead of (1), our simulations utilize the process model of an agent $u_{i}$ as

(25) $$\begin{array}{cc} u_{i}\left(k+1\right) & =u_{i}\left(k\right)+dt\times \left(s_{i}\left(k\right)\times c_{i}\left(k\right)\right)+n. \end{array}$$

Here, n is the process noise in the position of an agent. Variable n has a normal distribution with a mean of 0 and a standard deviation of 0.5 m. In this way, we simulate severe motion perturbation for each agent. For instance, sea current or unknown obstacles can generate motion perturbation for an agent. We added noise in the process model in order to show the robustness of the proposed controls against motion perturbation.

Considering the leader, there is no time delay in its control commands. For simulating flocking controls under communication delay, each follower $u_{j}$ (j<N) uses

(26) $$\begin{array}{cc} u_{j}\left(k+1\right) & =u_{j}\left(k\right)+dt\left(s_{N}\left(k-1\right)\times c_{N}\left(k-1\right)\right)+n. \end{array}$$

See that (26) considers the case where the communication delay between the leader $u_{N}$ and any other follower $u_{j}$ (j<N) is dt. This short time delay is feasible under the proposed formation controls since the leader and a follower can communicate within a single hop. Therefore, the control commands of $u_{N}$ at sampling instant k−1 are applied to $u_{j}$ (j<N) at sampling instant *k*.

We run 20 Monte-Carlo (MC) simulations to verify the robustness of the proposed multi-agent control. Initially, the leader is deployed at the origin. At the beginning of each MC simulation, the *i*-th agent (i∈{2,...,N}) is deployed randomly, utilizing the following equation.

(27) $$\begin{array}{c} u_{i}=\left(F^{R}\times rand-\frac{F^{R}}{2},F^{R}\times rand-\frac{F^{R}}{2},F^{R}\times rand-\frac{F^{R}}{2}\right). \end{array}$$

Here, rand returns a random number in the interval [0,1]. The simulation runs only when the initial position of each agent satisfies the initialNetworkAssumption.

In all MC simulations, the leader herds all agents to the goal successfully. This indicates that the flocking control works regardless of the initial agent deployment, as long as the initialNetworkAssumption is met initially. Whenever an agent collides with obstacles, the associated simulation ends. While we run 20 MC simulations, all agents safely reach the goal without colliding with obstacles.

### 5.1. Scenario 1

**Figure 2:**
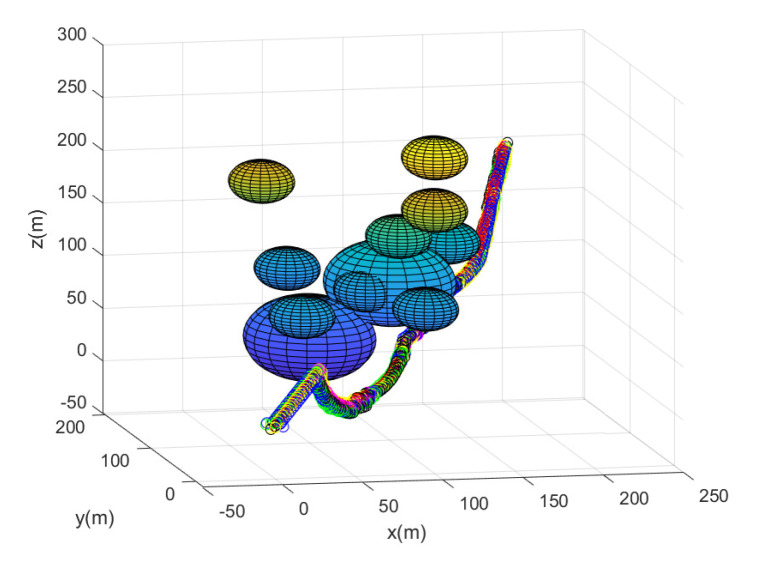
Scenario 1 with N=10 agents. The path of every agent until the leader reaches the goal. All obstacles are plotted with spheres. Every agent’s position at every 10 sampling instants is plotted with circles with distinct colors.

In this scenario, we use N=10 agents in total. Consider one MC simulation with the random deployment of every agent. Figure 2 shows the path of every agent until the leader reaches the goal. In this figure, the obstacles are plotted with spheres. Every agent’s position at every 10 sampling instants is plotted with circles with distinct colors. The leader is marked with a red diamond. All agents achieve collision avoidance with a spherical obstacle boundary while maintaining communication connectivity with the leader. All agents take 176 s to reach the goal. Running this scenario under MATLAB takes 40 s.

**Figure 3:**
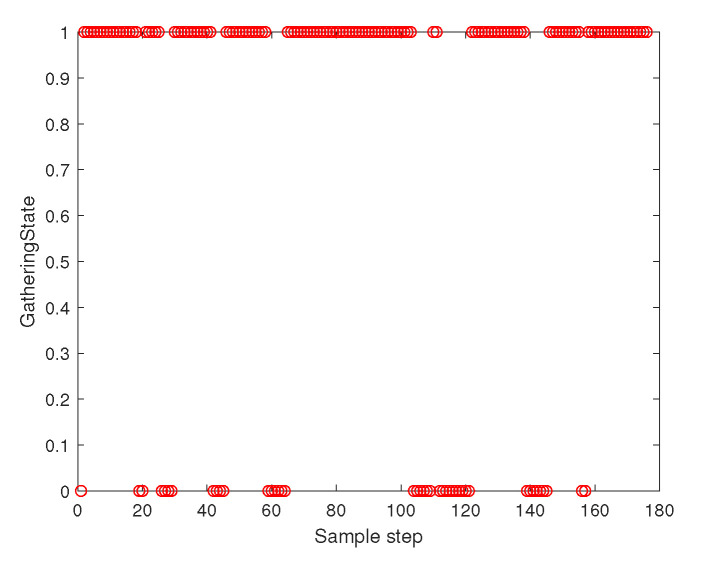
Scenario 1 with N=10 agents. The gathering state at each sampling instant *k*. As the gathering state is one, all followers are within the virtual sphere with radius $F^{R}\times \eta +\epsilon$. See that (2) is not satisfied occasionally. Whenever (2) is not satisfied, distributed gathering control in Section 4.2 is applied until (2) is met.

Figure 3 depicts the gathering state of the multi-agent system as time goes on. The gathering state at sampling instant *k* is one when (2) is satisfied at sampling instant *k*. The gathering state at sampling instant *k* is zero when (2) is not satisfied at sampling instant *k*. Figure 3 shows that (2) is not satisfied occasionally. Whenever (2) is not satisfied, distributed gathering control in Section 4.2 is applied until (2) is met.

**Figure 4:**
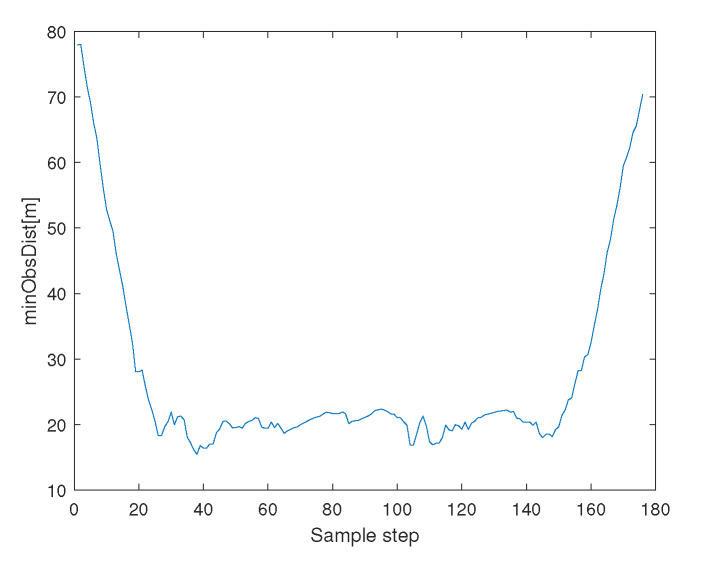
Scenario 1 with N=10 agents. This figure depicts the minimum distance between an agent and an obstacle boundary. See that collision avoidance is assured between every agent and an obstacle.

Figure 4 depicts the minimum distance between an agent and an obstacle boundary. See that collision avoidance is assured between every agent and an obstacle.

### 5.2. Scenario 2

**Figure 5:**
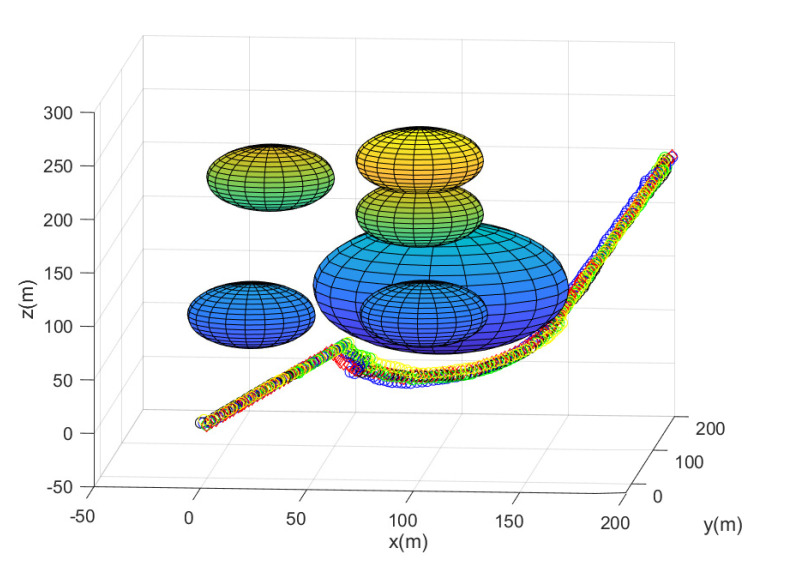
Scenario 2 with N=5 agents. Every agent’s position at every 10 sampling instants is plotted with circles with distinct colors.

**Figure 6:**
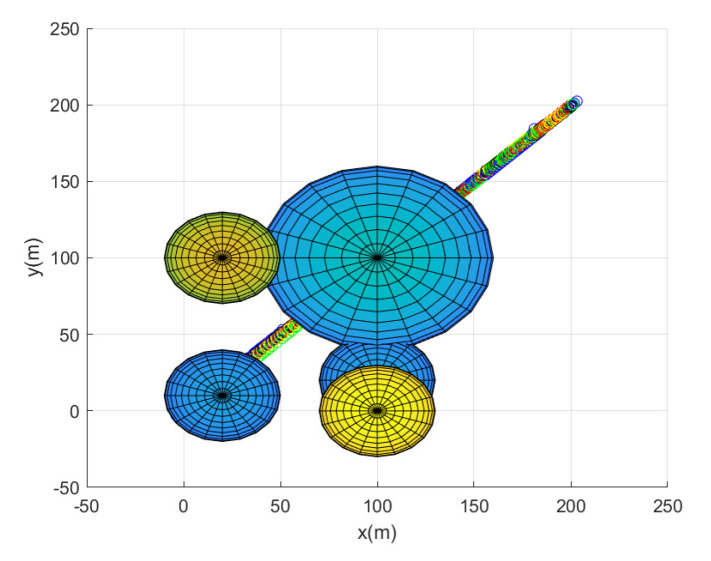
Scenario 2 with N=5 agents. The top view of Figure 5. See that the leader moves on the vertical plane, which is normal for the xy plane.

In this scenario, we deploy N=5 agents in total. Considering Scenario 2, Figure 5 shows the path of every agent until the leader reaches the goal. Figure 5 depicts the maneuver of every agent under the proposed flocking control. Every agent’s position at every 10 sampling instants is plotted with circles with distinct colors. Figure 6 depicts the top view of Figure 5. See that the leader moves on the vertical plane, which is normal for the xy plane.

All agents achieve collision avoidance with spherical obstacles while maintaining communication connectivity with the leader. All agents take 153 s to reach the goal. Running this scenario under MATLAB takes 5 s.

**Figure 7:**
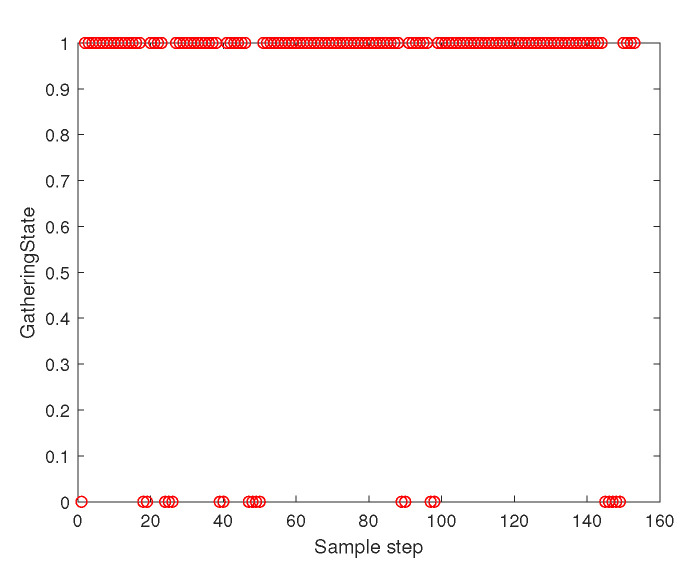
Scenario 2 with N=5 agents. The gathering state at each sampling instant *k*. As the gathering state is one, all followers are within the virtual sphere with radius $F^{R}\times \eta +\epsilon$. Whenever (2) is not satisfied, distributed gathering control in Section 4.2 is applied until (2) is met.

Figure 7 depicts the gathering state of the multi-agent system as time goes on. The gathering state at sampling instant *k* is one when (2) is satisfied at sampling instant *k*. The gathering state at sampling instant *k* is zero when (2) is not satisfied at sampling instant *k*. Figure 3 shows that (2) is not satisfied occasionally. Whenever (2) is not satisfied, distributed gathering control in Section 4.2 is applied until (2) is met.

**Figure 8:**
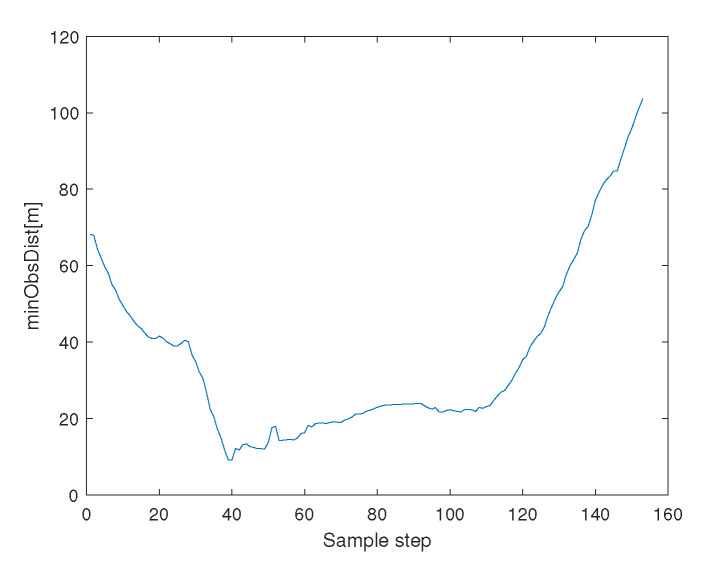
Scenario 2 with N=5 agents. This figure depicts the minimum distance between an agent and an obstacle boundary. See that collision avoidance is assured between every agent and an obstacle.

Figure 8 depicts the minimum distance between an agent and an obstacle boundary. See that collision avoidance is assured between every agent and an obstacle.

### 5.3. Scenario 3

In this scenario, we deploy N=7 agents in total. The 3D goal, which is only known to the leader, is located at [200,200,300].

**Figure 9:**
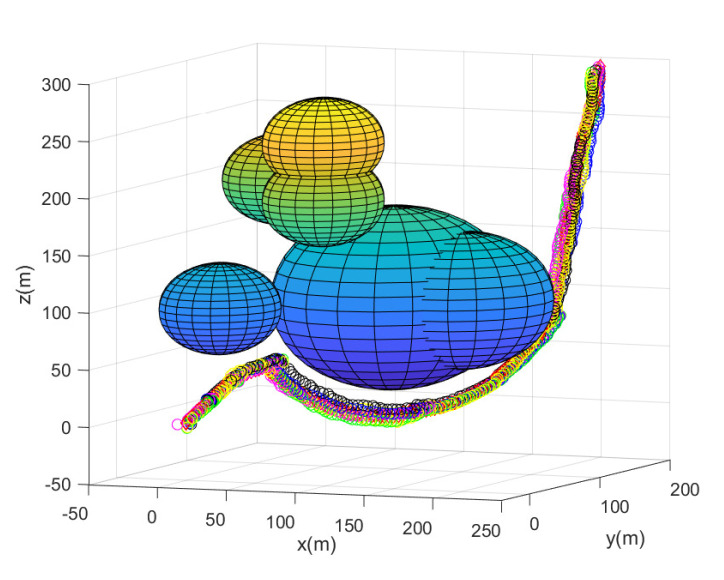
Scenario 3 with N=7 agents. Every agent’s position at every 10 sampling instants is plotted with circles with distinct colors.

**Figure 10:**
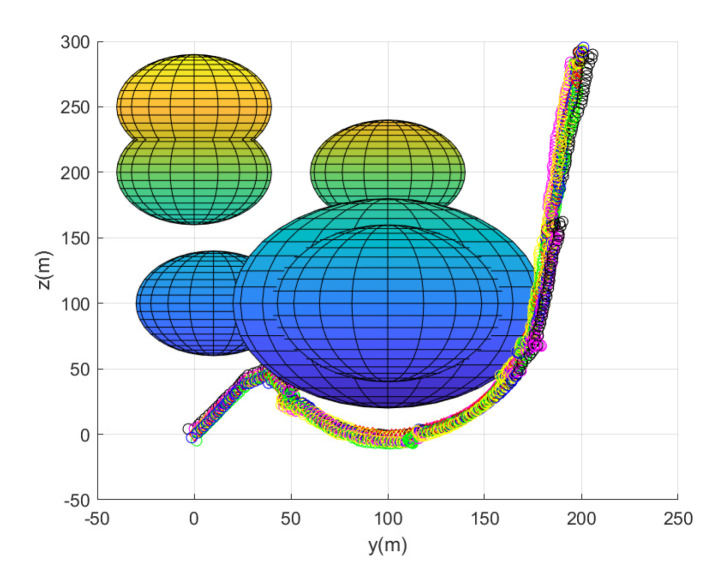
Scenario 3 with N=7 agents. The side view of Figure 9.

Considering Scenario 3, Figure 9 shows the path of every agent until the leader reaches the goal. Figure 9 depicts the maneuver of every agent under the proposed flocking control. Every agent’s position at every 10 sampling instants is plotted with circles with distinct colors. Figure 10 depicts the side view of Figure 9.

All agents achieve collision avoidance with obstacles while maintaining communication connectivity with the leader. All agents take 214 s to reach the goal. Running this scenario under MATLAB takes 21 s.

**Figure 11:**
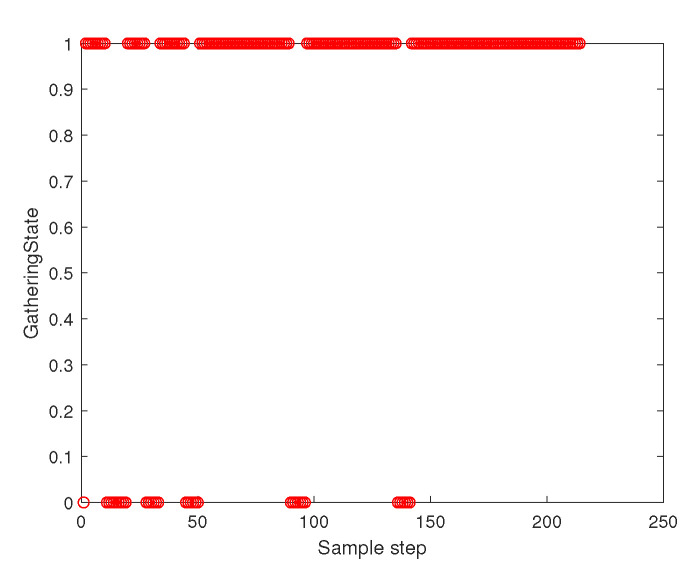
Scenario 3 with N=7 agents. The gathering state at each sampling instant *k*. As the gathering state is one, all followers are within the virtual sphere with radius $F^{R}\times \eta +\epsilon$. See that (2) is not satisfied occasionally. Whenever (2) is not satisfied, distributed gathering control in Section 4.2 is applied until (2) is met.

Figure 11 depicts the gathering state of the multi-agent system as time goes on. The gathering state at sampling instant *k* is one when (2) is satisfied at sampling instant *k*. The gathering state at sampling instant *k* is zero when (2) is not satisfied at the sampling instant *k*. Figure 3 shows that (2) is not satisfied occasionally. Whenever (2) is not satisfied, distributed gathering control in Section 4.2 is applied until (2) is met.

**Figure 12:**
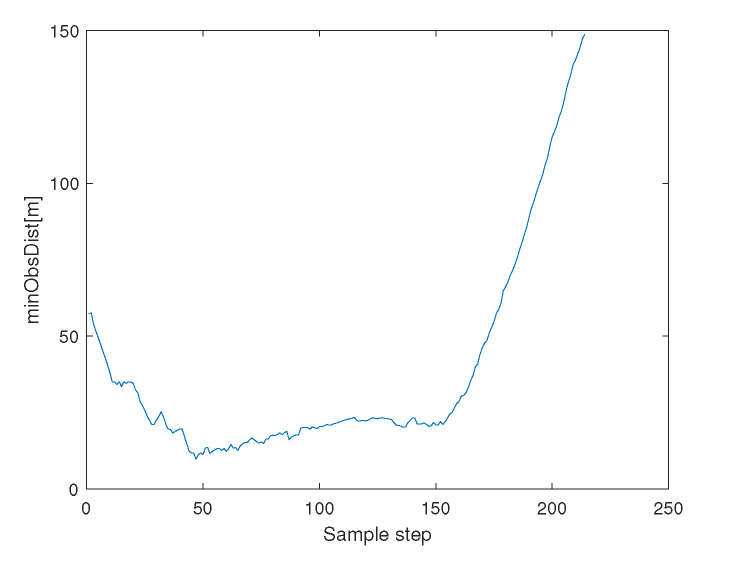
Scenario 3 with N=7 agents. This figure depicts the minimum distance between an agent and an obstacle boundary. See that collision avoidance is assured between every agent and an obstacle.

Figure 12 depicts the minimum distance between an agent and an obstacle boundary. See that collision avoidance is assured between every agent and an obstacle.

### 5.4. Scenario 4

In this scenario, we deploy N=3 agents in total. The 3D goal, which is only known to the leader, is located at [200,200,300]. In Scenario 4, small obstacles are distributed in the 3D workspace.

**Figure 13:**
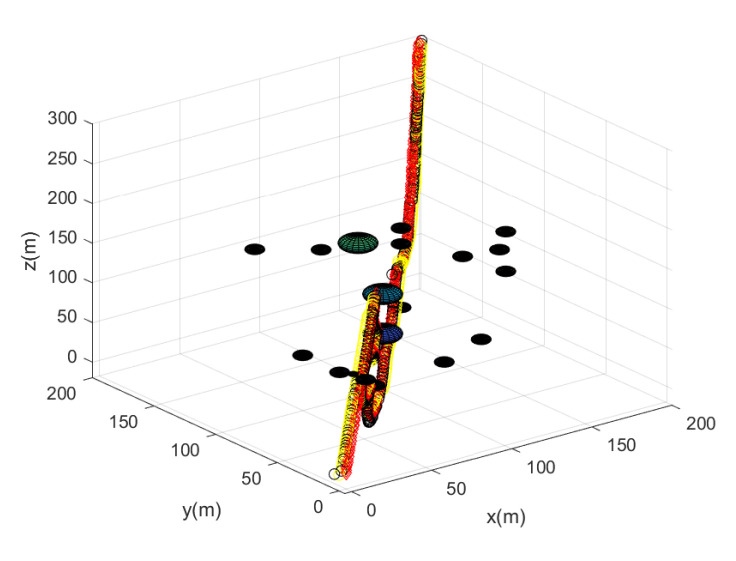
Scenario 4 with N=3 agents. Every agent’s position at every 10 sampling instants is plotted with circles with distinct colors.

**Figure 14:**
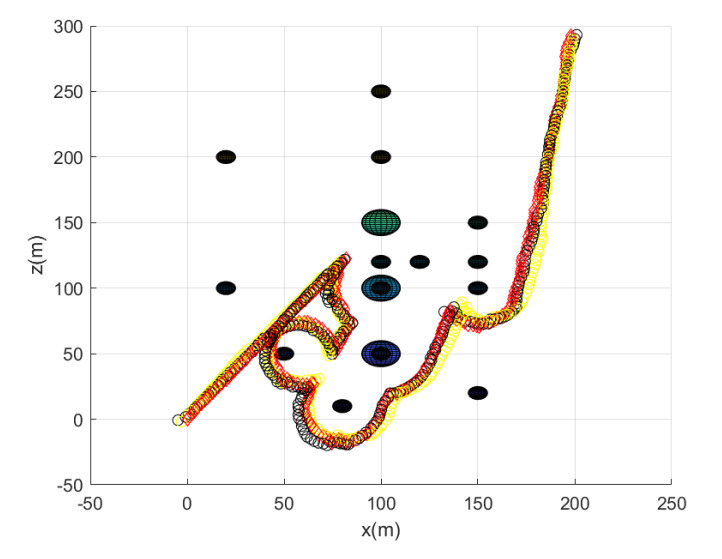
Scenario 4 with N=3 agents. The side view of Figure 13.

Considering Scenario 4, Figure 13 shows the path of every agent until the leader reaches the goal. Figure 13 depicts the maneuver of every agent under the proposed flocking control. Every agent’s position at every 10 sampling instants is plotted with circles with distinct colors. Figure 14 depicts the side view of Figure 13.

All agents achieve collision avoidance with obstacles while maintaining communication connectivity with the leader. All agents consume 300 s to reach the goal. Running this scenario under MATLAB takes 8 s.

**Figure 15:**
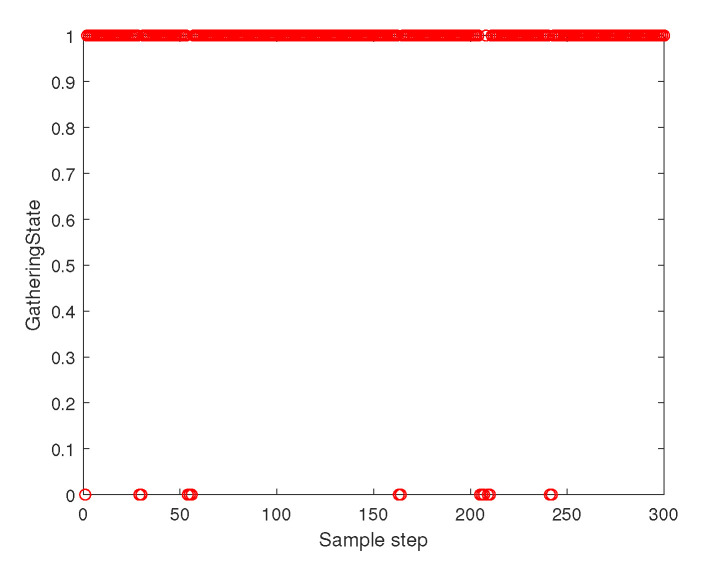
Scenario 4 with N=3 agents. The gathering state at each sampling instant *k*. As the gathering state is one, all followers are within the virtual sphere with radius $F^{R}\times \eta +\epsilon$. See that (2) is not satisfied occasionally. Whenever (2) is not satisfied, distributed gathering control in Section 4.2 is applied until (2) is met.

Figure 15 depicts the gathering state of the multi-agent system as time goes on. The gathering state at sampling instant *k* is one when (2) is satisfied at sampling instant *k*. Figure 15 shows that (2) is not satisfied occasionally. Whenever (2) is not satisfied, the distributed gathering control in Section 4.2 is applied until (2) is met.

**Figure 16:**
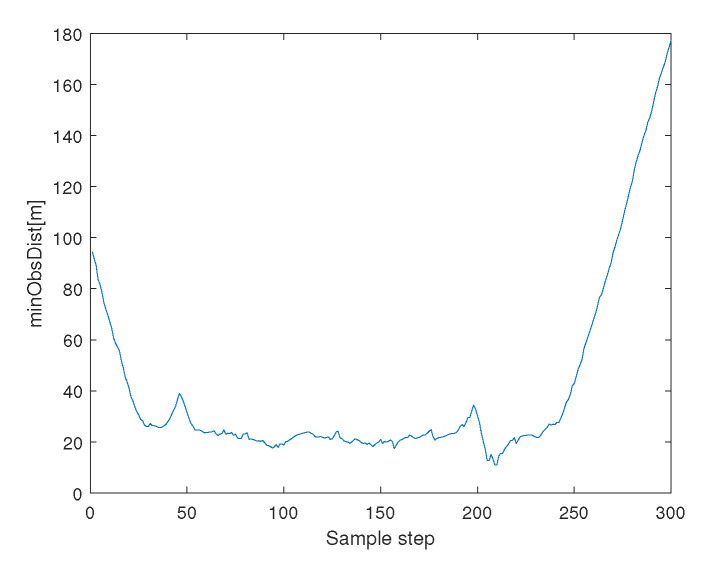
Scenario 4 with N=3 agents. This figure depicts the minimum distance between an agent and an obstacle boundary. See that collision avoidance is assured between every agent and an obstacle.

Figure 16 depicts the minimum distance between an agent and an obstacle boundary. See that collision avoidance is assured between every agent and an obstacle.

## 6. Conclusions

In this paper, we develop underwater flocking controls such that the leader can herd all agents to the goal safely while preserving communication connectivity with the leader. As far as we know, our article is novel in developing underwater flocking controls based on a single leader so that a swarm of agents can safely flock to its goal in a priori unknown cluttered underwater environments. The proposed flocking controls can be applied to various robots, such as ground robots, aerial robots or underwater robots. In the future, the effectiveness of the proposed flocking controls will be demonstrated by utilizing experiments with real robots.

In practice, agents need to avoid moving obstacles while they move. Reactive collision avoidance methods in [49,50] can be integrated with the proposed flocking controls so that an agent can avoid collision with moving obstacles. This reactive collision avoidance requires that an agent has local sensors for sensing a nearby obstacle.

We acknowledge that our swarm controls are not robust to leader failures. Only the leader can access the global coordinates of the goal point. Furthermore, only the leader can localize itself utilizing various localization approaches. Thus, once the leader fails, not all agents can reach the goal. In practice, the followers surrounding the leader can be utilized to protect the leader from enemy attacks. This is similar to a bee colony, where the queen bee is protected by all the other bees.

There may be a case where the robot swarm needs to move through a narrow tunnel. In this case, we can further decrease the swarm’s volume by re-running the distributed gathering controls in Section 4.2. We can decrease the swarm’s formation volume by decreasing η in (2). In the future, we will handle flocking controls by considering the cases where the robot swarm moves in various environments, such as narrow tunnels.

This paper considered a spherical formation such that all followers are gathered close to the leader. Reference [51] addressed a distributed 3D algorithm for coordinating a swarm of robots to spatially self-aggregate into an arbitrary shape based on only local interactions. We can change the formation shape by applying the distributed 3D algorithm in [51]. Once the robot formation changes to a desired one, we can apply the proposed flocking controls so that the leader can herd all robots to the goal safely. In the future, we will tackle flocking controls based on a formation with an arbitrary shape.

## Data Availability

Not applicable.
